# Syndromic and sporadic inflammatory/hyperplastic small-bowel polyps: a comparative study

**DOI:** 10.1093/gastro/gov020

**Published:** 2015-06-06

**Authors:** Xiuli Liu, Derrick Chen, Mohannad Dugum, Bela Horvath, Lisi Yuan, Shu-Yuan Xiao

**Affiliations:** ^1^Department of Anatomic Pathology, Cleveland Clinic, Cleveland, OH, USA; ^2^Department of Internal Medicine, Cleveland Clinic, Cleveland, OH, USA; ^3^Department of Pathology, University of Chicago, IL, USA

**Keywords:** hyperplastic polyp, inflammatory polyp, small bowel, juvenile polyp, polyposis

## Abstract

**Background:** Inflammatory/hyperplastic small-bowel polyps (SBPs) occur either sporadically or in patients with a polyposis syndrome; however, comparison between these two settings of the histological features of SBPs has not been reported and the etiology of sporadic inflammatory/hyperplastic SBPs remains unclear.

**Method:** Twenty-eight cases of sporadic inflammatory/hyperplastic SBPs and nine cases of syndromic SBPs were retrieved from the Department of Anatomic Pathology at the Cleveland Clinic. Clinico-demographics and histological features were compared between the two groups.

**Results:** Patients with syndromic inflammatory/hyperplastic SBPs were younger (48 *vs.* 63 years; *P = *0.007) and had higher rates of hemorrhagic telangiectasia (55.6% *vs.* 0%; *P = *0.000), gastric polyps (87.5% *vs.* 21.4%; *P = *0.001), and family history of colon cancer (62.5% *vs.* 11.1%; *P = *0.014). Sporadic cases were more frequently associated with gastro-esophageal reflux (35.7% *vs.* 0%; *P = *0.079) and anti-reflux medication use (55.6% *vs.* 11.1%; *P = *0.026). Histologically, the syndromic SBPs were more often of pure intestinal type (45.4% *vs.* 3.8%; *P = *0.005) and had prominent vessels (81.8% *vs.* 42.3%; *P = *0.036).

**Conclusions:** Patients with syndromic SBPs are younger and have higher rates of hemorrhagic telangiectasia, gastric polyps, and family history of colon cancer. Histologically, syndromic inflammatory/hyperplastic SBPs are more likely to be of pure intestinal type and to have prominent vessels.

## Introduction

Colorectal hyperplastic and inflammatory polyps are common and well-characterized. In contrast, inflammatory and/or hyperplastic small-bowel polyps (SBPs) occur less frequently and have not been so well studied.

Patients with juvenile polyposis syndrome (JPS) and other polyposis syndromes are prone to developing inflammatory polyps in the upper gastro-intestinal tract and gastro-intestinal cancer [1, 2]. *SMAD4* (DPC4) knockout mice develop multiple duodenal and gastric polyps that demonstrate considerable similarity to those found in human JPS, including moderate stromal cell proliferation and infiltration by plasma cells and eosinophils [2]. In addition, patients with Cowden syndrome may also develop hyperplastic or hamartomatous polyps in the gastro-intestinal tract including the stomach, duodenum, and colorectum [3, 4]. Further, some patients with *MYH*-associated polyposis have hyperplastic polyps in the colon and rectum [5, 6]. While the colon polyps associated with these syndromes are recognized and have been studied, their counterparts in the small bowel have not.

Sporadic—or non-syndromic—hyperplastic polyps of the duodenum have previously been described [7]. In this study, involving a small case series, the authors reported that these polyps occur more frequently in the second part of the duodenum and are accompanied by upper gastro-intestinal tract disease, including Barrett’s esophagus (BE), *Helicobacter* gastritis, and mild chronic gastritis, in 77.8% of cases.

Comparison, between syndromic and sporadic inflammatory/hyperplastic SBPs, of histological features has not been previously reported and the etiology of sporadic inflammatory/hyperplastic SBPs remains unclear. The purpose of the present study is to compare the histology of inflammatory/hyperplastic SBPs in the sporadic and syndromic settings and to identify clinico-demographic features and concurrent diseases associated with sporadic inflammatory/hyperplastic SBPs.

## Materials and Methods

### Study population

The database of the Department of Anatomic Pathology at the Cleveland Clinic, from 2004 through 2013, was searched for “gastric foveolar hyperplasia”, “inflammatory polyp”, and “hyperplastic polyp” in the duodenum, jejunum, or ileum. The search found a total of twenty-eight cases, which were designated as the sporadic inflammatory/hyperplastic SBP group. Details of nine cases with syndromic SBPs [JPS (*n = *6), Cowden disease (*n = *1), *MYH*-associated polyposis (*n = *1), and familial adenomatous polyposis (*n = *1)] were retrieved from the same pathology database during the same period. All cases with syndromic inflammatory/hyperplastic SBPs were confirmed by genetic testing. This study was approved by the Institutional Review Board at the Cleveland Clinic.

### Clinico-demographic information and endoscopic findings

The patients' medical charts were reviewed for demographics (age and gender), presence of hemorrhagic telangiectasia, presence of gastric polyps (inflammatory, hyperplastic, or fundic gland polyp), gastro-esophageal reflux diagnosis, medications [non-steroid anti-inflammatory drugs (NSAIDs) and anti-reflux agents], presence of anemia (defined as hemoglobin less than 11 g/dL for women and less than 13 g/dL for men), personal history of malignancy, family history of colon cancer, and family history of stomach cancer. The numbers and locations of polyps were retrieved from endoscopic reports.

### Histological review

Slides, for histological review, were available for twenty-six sporadic SBPs and 11 syndromic SBPs. Blinded to the clinical information, a gastro-intestinal pathologist re-evaluated all polyps. Histological features examined included the type of lesional epithelium (intestinal, gastric, and mixed intestinal/gastric), the presence of epithelial hyperplasia (serration of any crypt/gland), cystic dilation of any crypts/glands, stromal overgrowth (having a stoma-to-epithelium ratio greater than one), stromal edema, erosion, acute inflammation (presence of neutrophilic inflammation in the *lamina propria* and/or epithelium), chronic inflammation (presence of mononuclear inflammation expanding the *lamina propria*), Brunner's gland hyperplasia, and prominent vessels (the presence of more than one dilated vessel in the lesion). Some of the studied features are based on, derived from or modified from histological features of hamartomatous colonic or gastric polyps from patients with JPS or upper gastro-intestinal tract polyps in *SMAD4*-mutated mice [2, 8–12].

### Statistical analysis

Continuous variables were summarized as mean and standard deviation (SD). Categorical variables were summarized as count and proportion, and compared using Fisher’s exact test. Continuous variables were described as mean plus or minus standard deviation, and compared using Student’s *t*-test. A *P*-value of less than 0.05 was considered statistically significant. Statistical analysis was performed using ‘R’, version 2.15.2 (R Development Core Team, 2012, Vienna, Austria).

## Results

### Clinical and demographic characteristics

This study included 9 patients with syndromic inflammatory/hyperplastic SBP and 28 cases of sporadic inflammatory/hyperplastic SBPs. All cases with syndromic inflammatory/hyperplastic SBPs were confirmed by genetic testing (Table 1). Six of nine cases (66.7%) of syndromic SBPs had a personal history of colon polyps, and two of nine (22.2%) had had a previous gastrectomy for polyposis. Four of 28 patients (14.3%) from the sporadic group had cirrhosis, and 2 of 28 (7.1%) had chronic renal failure.

**Table 1.** Genetic abnormalities in patients with syndromic inflammatory/hyperplastic small-bowel polyps

Syndromic cases	Gene affected	Abnormality	Final diagnosis
1	*SMAD4*	Ex2_3,5_12 dup	Juvenile polyposis syndrome
2	*SMAD4*	C.1139 G>A	Juvenile polyposis syndrome
3	*PTEN*	A deleterious mutation which includes a full gene deletion (identified by *PTEN* gene deletion and large re-arrangement analysis)	Cowden syndrome
4	*MYH*	Homozygous for MYH mutation (G396D)	MYH-associated polyposis
5	*APC*	A deletion of the 5q22.1q22.2 region, encompassing the *APC* gene	Familial adenomatous polyposis
6	*SMAD4*	Exon 8 duplication	Juvenile polyposis syndrome
7	*SMAD4*	*SMAD4* mutation, not further specified	Juvenile polyposis syndrome
8	*PTEN, BMPR1A*	Large 10q deletion which includes *PTEN* and *BMPR1A* (identified by karyotype analysis)	Juvenile polyposis syndrome
9	*SMAD4*	*SMAD4* mutation, not further specified	Juvenile polyposis syndrome

As shown in Table 2, patients with syndromic inflammatory/hyperplastic SBPs were younger than those with sporadic SBPs (48 *vs*.** 63 years in mean age; *P = *0.007). Syndromic patients had higher rates of hemorrhagic telangiectasia (55.6% *vs.* 0%; *P = *0.000), gastric polyps (87.5% *vs.* 21.4%; *P = *0.001), and family history of colon cancer (62.5% *vs.* 11.1%; *P = *0.014). Conversely, sporadic cases were more frequently associated with a clinical diagnosis of gastro-esophageal reflux (35.7% *vs.* 0%; *P = *0.079) and use of anti-reflux medication (55.5% *vs.* 11.1%; *P = *0.026). Other examined clinical features, including use of NSAIDs (11.1% *vs.* 29.6%), anemia (33.3% *vs.* 50.0%), personal malignancy history (22.2% *vs.* 11.1%), and family history of stomach cancer (12.5% *vs.* 5.6%) showed no statistically significant difference between the syndromic and sporadic groups.

**Table 2.** Comparison of demographics and clinical information between patients with syndromic *vs.* sporadic inflammatory/hyperplastic small-bowel polyps.

Characteristics	Patients with syndromic SBPs (*n = *9)	Patients with sporadic SBPs (*n = *28)	*P-value*
Age, years	48 ± 15	63 ± 12	0.007
Males, *n* (%)	3 (33.3)	15 (53.6)	0.447
Hemorrhagic telangiectasia, *n* (%)	5 (55.6)	0 (0.0)	0.000
Anemia, *n* (%)	3 (33.3)	14 (50.0)	0.462
Gastric polyps, *n* (%)	7 (87.5)^a^	6 (21.4)	0.001
Reflux, *n* (%)	0 (0.0)	10 (35.7)	0.079
Anti-reflux medication use, *n* (%)	1 (11.1)	15 (55.6)^a^	0.026
NSAID use, *n* (%)	1 (11.1)	5 (18.5)^a^	1
Personal malignancy history, *n* (%)	2 (22.2)	2 (11.1)^a^	0.582
Family history of colon cancer, *n* (%)	5 (62.5)^a^	2 (11.1)^a^	0.014
Family history of stomach cancer, *n* (%)	1 (12.5)^a^	1 (5.6)^a^	0.529

^a^Information on some patients was not available.

NSAID=non-steroid anti-inflammatory drug; SBP = small-bowel polyp

### Concurrent gastric disease

Twenty-seven (96.4%) of the 28 patients with sporadic inflammatory/hyperplastic SBPs had endoscopic examination and biopsy of the stomach. As shown in Table 3, concurrent gastric disease was found in 18 patients (66.7%), including reactive gastropathy (22.2%), gastritis (22.2%, including four non-*Helicobacter* gastritis, one atrophic gastritis, and one *Helicobacter* gastritis), gastric polyps (22.2%, including five fundic gland polyps, and one hyperplastic polyp), and hypertensive gastropathy (7.4%). Two patients with syndromic inflammatory/hyperplastic SBPs had gastrectomy for gastric polyposis and one had no information on gastric examination. Five of the remaining six patients (83.3%) had concurrent hyperplastic polyps (three cases of juvenile polyposis and one of Cowden disease) and innumerable fundic gland polyps (the case of familial adenomatous polyposis). The patient with *MYH*-associated polyposis had a normal stomach.

**Table 3.** Comparison of the frequency of concurrent gastro-esophageal disease in patients with syndromic and sporadic inflammatory/hyperplastic small-bowel polyps

Concurrent gastro-esophageal disease	Patients with syndromic SBPs (*n = *7)	Patients with sporadic SBPs (*n = *27)	*P-value*
Gastric disease, *n* (%)	5 (83.3)^a^	18 (66.7)	0.64
Reactive gastropathy	0 (0.0)	6 (22.2)	0.563
Gastritis	0 (0.0)	6 (22.2)	0.563
Gastric polyp	5 (83.3)	6 (22.2)	0.010
Hypertensive gastropathy	0 (0.0%)	2 (7.4)	1
Esophageal disease, *n* (%)	3 (42.9)	17 (63.0)	0.41
Esophagitis/Barrett’s esophagus	1 (14.3)	5 (18.5)	1
Varices	0 (0.0)	4 (14.8)	0.559
Hiatal hernia	2 (28.6)	9 (33.3)	1
Esophageal ring	0 (0.0)	2 (7.4)	1

^a^There was no information on gastric examination for one patient

SBP = small-bowel polyp

### Concurrent esophageal disease

Twenty-seven patients with sporadic inflammatory/hyperplastic SBPs (96.4%) had had endoscopic examination and biopsies of the esophagus. As shown in Table 3, concurrent esophageal disease was seen in 17 patients (63.0%), and included reflux esophagitis (14.8%), Barrett’s esophagus (3.7%), hiatal hernia (33.3%), varices (14.8%), and esophageal ring (7.4%). Seven of nine patients with syndromic inflammatory/hyperplastic SBPs (77.7%) had had esophageal examination and biopsies, and concurrent esophageal disease was noted in three cases (42.9%): one (the patient with Cowden disease) had esophagitis with innumerable nodules 1–2 mm in size along the entire esophagus and two cases showed hiatal hernia.

### Endoscopic and histological findings

Endoscopically the duodenal lesions were described as “small polyp”, “small nodule” or, rarely, “prominent ampulla”. The polyp size was only documented in a few cases, insufficient for further analysis. The number of polyps was reported by the endoscopist as being single, few, or multiple; for the purpose of comparison, this enumeration was simplified into two categories: single (one polyp) or multiple (two or more polyps). A multiplicity of polyps was seen in 75.0% of syndromic patients *vs.* 42.6% of sporadic cases (*P = *0.23). For the syndromic group, the location of the polyp was in the duodenal bulb for two of eight cases (25.0%) and in other sites of the small bowel for six cases [the second part of the duodenum (*n = *2), an unspecified site of the duodenum (*n = *3), and the jejunum (*n = *1)]. For the sporadic group, the location of the polyp was in the duodenal bulb for 15 of 28 cases (53.5%) and in other sites of the small bowel for 13 cases [the second part of the duodenum (*n = *7), an unspecified site the duodenum (*n = *4), and the proximal ileum (*n = *1)].

**Figure 1.**
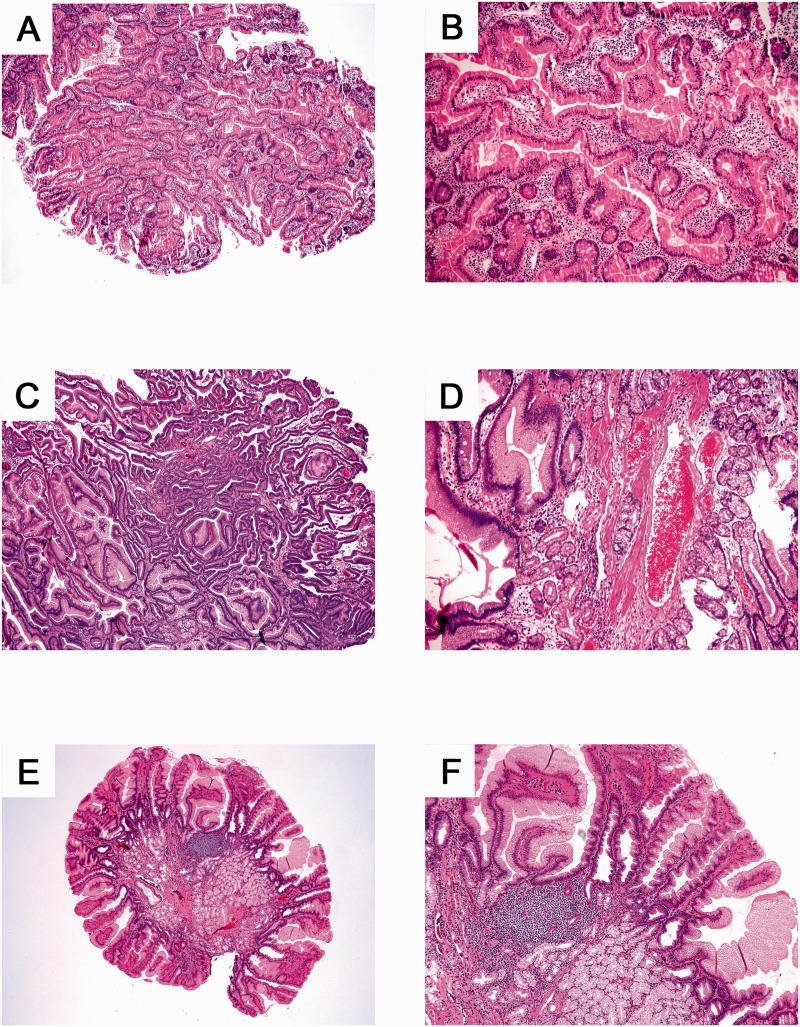
Histological features of inflammatory/hyperplastic small-bowel polyps (SBPs) in syndromic and sporadic cases. (A, B) Intestinal-type hyperplastic SBP in a patient with juvenile polyposis syndrome. (C, D) Prominent vessel within gastric-type hyperplastic SBP in a patient with juvenile polyposis syndrome. (E, F) Sporadic gastric-type hyperplastic SBP.

Twenty-six separately submitted polyps from 26 patients with sporadic SBPs and 11 separately submitted SBPs from eight syndromic patients were subjected to histological review for the features described in the Methods section. As shown in Table 4, the syndromic inflammatory/hyperplastic SBPs were more often of pure intestinal type (45.4% *vs.* 3.8%; *P = *0.005) (Figure 1, A & B). Prominent vessels were more likely to be present in syndromic than in sporadic inflammatory/hyperplastic SBPs (81.8% *vs.* 42.3%; *P = *0.036) (Figure 1, C–F). The frequency of other examined histological features—including epithelial hyperplasia/serration, cystic dilation of glands, stromal overgrowth, stromal edema, surface erosion, acute inflammation, chronic inflammation, and Brunner’s gland hyperplasia—was not significantly different between the two groups (Table 4).

**Table 4.** Comparison of histological features of syndromic and sporadic inflammatory/hyperplastic small-bowel polyps

Histological features	Syndromic inflammatory/hyperplastic SBPs (*n = *11)	Sporadic inflammatory/hyperplastic SBPs (*n = *26)	*P*-value
Pure intestinal type (%)	5 (45.4)	1 (3.8)	0.005
Epithelial hyperplasia (%)	8 (72.3)	23 (88.5)	0.335
Cystic dilation of glands (%)	4 (36.4)	11 (42.3)	1
Stromal overgrowth (%)	5 (45.5)	13 (50.0)	1
Stromal edema (%)	4 (36.4)	8 (30.8)	1
Surface erosion (%)	2 (18.2)	8 (30.8)	0.688
Acute inflammation (%)	5 (45.5)	11 (42.3)	1
Chronic inflammation (%)	10 (90.9)	23 (88.5)	1
Brunner’s gland hyperplasia (%)	5 (45.4)	16 (61.5)	0.475
Prominent vessels (%)	9 (81.8)	11 (42.3)	0.036

SBP = small-bowel polyp.

## Discussion

Benign inflammatory/hyperplastic polyps of the small bowel have only rarely been studied and described in the literature [7, 13, 14]. Although duodenal and gastric inflammatory/hyperplastic polyps occur frequently in familial JPS and may histologically resemble hyperplastic polyps or inflammatory polyps in many cases [9, 13], a direct histological comparison between sporadic inflammatory/hyperplastic SBPs and syndromic inflammatory/hyperplastic SPBs has not previously been reported. The current study revealed that polyps of pure intestinal epithelium and the presence of prominent vessels are more frequently seen in syndromic inflammatory/hyperplastic SBPs. The high frequency of prominent vessels in syndromic inflammatory/hyperplastic SPBs seen in this study is consistent with the finding of increased mucosal microcirculation in juvenile polyps in children from a previous report [15]. This study also showed marked histomorphological overlap between syndromic and sporadic SBPs; the frequency of stromal changes (either overgrowth or edema), inflammation (acute or chronic), surface erosion, architecture distortion (hyperplasia/serration or cystic dilation), and Brunner’s gland hyperplasia was similar in both entities. These results suggest significant histological overlap of juvenile (the presumed “hamartomatous”) SBP with hyperplastic and inflammatory polyps, and are consistent with previous reports of significant histological overlapping among hyperplastic polyp, juvenile polyp, and inflammatory polyp of the colorectum [10, 11], and the similarity between juvenile polyp and hyperplastic polyp of the stomach [9].

Polyp location and the frequency of multiplicity showed no statistically significant difference between the syndromic and sporadic inflammatory/hyperplastic SBPs. In the current study, only 25% of sporadic inflammatory/hyperplastic SBPs occurred in the second part of the duodenum, fewer than the previous reported rate of 55.5% [7]. This discrepancy may be due to the relatively small size of their series (9 *vs.* 28 cases in this study).

Interestingly, gastric epithelial differentiation was found in 54.6% of syndromic SBPs. This finding is consistent with a previous report, in which a juvenile polyp with *SMAD4* mutation showed gastric differentiation (defined by expression of MUC5AC protein) [8]. Similarly, 96% of sporadic inflammatory/hyperplastic SBPs had gastric-type epithelium (either pure or mixed with intestinal epithelium) as well, which is consistent with a previous report that gastric differentiation occurred in 89% of hyperplastic polyps of the duodenum [7].

Our study revealed that patients with syndromic inflammatory/hyperplastic SBPs were younger, had higher rates of concurrent gastric polyps (83.3% *vs.* 22.2%; *P = *0.001), and were more likely to have a family history of colon cancer (62.5% *vs.* 11.1%; *P = *0.014). Notably, our study revealed that hemorrhagic telangiectasia only occurred in syndromic patients (55.6% *vs.* 0%; *P = *0.000). These results suggest that inflammatory/hyperplastic SBPs should be interpreted within the appropriate clinical context; specifically, the results emphasize the need to synthesize information on hemorrhagic telangiectasia, concurrent gastric polyp, personal history of colon polyps, and family history of colon cancer in triaging patients with inflammatory/hyperplastic SBPs for genetic counseling and further genetic testing. Among these factors, telangiectasia—a readily identifiable clinical manifestation—is specific to syndromic cases and should be sought clinically in patients of young or middle age who have multiple inflammatory/hyperplastic SBPs.

This study also revealed that sporadic cases were more frequently associated with a clinical diagnosis of gastro-esophageal reflux (35.7% *vs.* 0%; *P = *0.079) and use of anti-reflux medication (55.6% *vs.* 11.1%; *P = *0.026); however, the frequency of concurrent esophagitis/BE in patients with sporadic inflammatory/hyperplastic SBP is not significantly different from the syndromic group (18.5% *vs.* 14.3%; *P = *1.0). Although these results suggest that sporadic inflammatory/hyperplastic SBPs may be associated with relatively early peptic injury—prior to the development of histologically evident esophagitis—the observed associations could also merely reflect the fact that Gastroesophageal reflux disease (GERD) is a common indication for esophago-gastroduodenoscopy (EGD) in sporadic cases. The frequency of BE in patients with sporadic inflammatory/hyperplastic SBP in this study was only 3.7%, which was significantly lower than the previously reported 55.6% rate of BE in patients with hyperplastic polyps of the duodenum [7]. This discrepancy is probably due to more stringent diagnostic criteria used for BE in the United States. Interestingly, the frequency of use of NSAIDs was not significantly different between the two groups, suggesting that NSAIDs may not play a significant role in the development of sporadic inflammatory/hyperplastic SBPs. The frequency of gastritis in the sporadic inflammatory/hyperplastic SBPs group was 22.2%, consistent with the previous report that 22.2% of patients with hyperplastic polyp of the duodenum had gastritis [7]. The lack of a significant difference in the family history of stomach cancer between the syndromic and sporadic inflammatory/hyperplastic SBP groups (12.5% *vs.* 5.6%; *P = *0.529) may be due to their small sample size.

There are several limitations to the present study: the data are limited by the availability and varying quality of clinical information present within the medical records. For example, many endoscopic reports did not have detailed information on the sizes, number, and locations of polyps. The majority of patients with sporadic inflammatory/hyperplastic SBPs did not undergo colonoscopy, thus preventing direct comparison of frequency and burden of colon polyps between the two groups. While all syndromic cases of inflammatory/hyperplastic SBPs were confirmed by appropriate genetic testing, the study group was presumed to be sporadic without confirmatory genetic testing. Also, the number of cases in this study set was small, and the selection of cases was from a patient population seen at a highly specialized tertiary care center, so conclusions from this study may not be generally applicable to other practice settings.

In summary, this study has revealed a significant histological overlap between sporadic and syndromic inflammatory/hyperplastic SBPs. It highlights the importance of synthesizing patient age, clinical information (telangiectasia, presence of concurrent gastric polyps, and personal history of colon polyps), and family history of colorectal cancer when triaging patients with inflammatory/hyperplastic SBPs for genetic counseling and testing for polyposis syndromes. Large studies are needed to confirm these findings and to determine the natural history of sporadic inflammatory/hyperplastic SBPs.

## Author contributions

Xiuli Liu designed the research study, performed the research, analysed the data, and co-wrote the paper. Derrick Chen analysed the data and co-wrote the paper. Mohannad Dugum performed the research and co-wrote the paper. Bela Horvath performed the research, analysed the data, and co-wrote the paper. Lisi Yuan analysed the data and co-wrote the paper. Shu-Yuan Xiao designed the research study.

*Conflict of interest statement*: none declared.
